# Indole at low concentration helps exponentially growing *Escherichia coli* survive at high temperature

**DOI:** 10.1371/journal.pone.0188853

**Published:** 2017-12-07

**Authors:** Junyan Liu, David Summers

**Affiliations:** Department of Genetics, University of Cambridge, Cambridge, United Kingdom; University of Manchester, UNITED KINGDOM

## Abstract

A culture of stationary phase *Escherichia coli* cells has been reported to produce copious indole when exposed to high temperature (50°C), and this response has been proposed to aid survival. We reinvestigated this phenomenon and found that indole production under these conditions is probably not a direct response to heat stress. Rather, *E*. *coli* produces indole when growth is prevented, irrespective of whether this is due to heat stress, antibiotic treatment or the removal of nutrients. Moreover, 300μM indole produced at 50°C does not improve the viability of heat stressed cells. Interestingly, a much lower concentration of indole (20 μM) improves the survival of an indole-negative strain (*ΔtnaA*) when heat stressed during exponential growth. In addition we have shown that the distribution of tryptophanase, the enzyme responsible for indole synthesis, is highly heterogeneous among cells in a population, except during the transition between exponential and stationary phases. The observation that, despite the presence of the tryptophanase, very little indole is produced during early exponential phase suggests that there is post-translational regulation of the enzyme.

## Introduction

Indole was discovered over one hundred years ago and has long been a signature metabolite in the diagnosis of *Escherichia coli* infection [1]. In *E*. *coli* indole is produced from tryptophan by the enzyme tryptophanase (TnaA), generating pyruvate and ammonia in the same reaction [2]. Tryptophanase is encoded by the *tnaCAB* operon that is regulated by catabolite repression [3] and transcription anti-termination [4, 5]. Transcription of the tryptophanase operon initiates from a CAP-dependent promoter which is activated as cAMP builds up [3]. The progression of transcription into the structural gene region requires the presence of exogenous tryptophan, detected by a *tnaC*-dependent mechanism, to stop Rho-dependent termination before RNA polymerase reaches *tnaA* and *tnaB* [6, 7].

There is growing evidence that this simple regulatory picture is far from complete. For example, the stationary phase sigma factor, σ^S^ (*rpoS*), is essential for normal expression of TnaA [8] and another transcription factor, MarA, can also increase TnaA expression [9]. Furthermore, the response regulator TorR can potentially bind the *tna* promoter and may be responsible for TnaA induction in the presence of trimethylamine N-oxide (TMAO) under anaerobic conditions [10]. Post-translational regulation of tryptophanase activity also plays a role since the non-coding RNA Rcd, transcribed from dimerised ColE1 plasmids [11], increases indole production by TnaA [12]. Li and Young [13] have presented preliminary evidence for post-translational regulation of TnaA activity in plasmid-free cells. They found that when glucose or arabinose was added to a culture during the transition from exponential to stationary phase (the time when indole is being synthesized rapidly) indole production was halted immediately. This suggests that carbohydrates inactivate TnaA, although the authors conclude that the effect is likely to be indirect as neither glucose nor arabinose interacts directly with TnaA *in vitro* [13].

Over the past decade indole has attracted increasing interest from researchers who have described a wide range of potential functions for the molecule [1, 14, 15]. Indole reduces *E*. *coli* motility and has knock-on effects on biofilm formation [16]. It can block cell division by reducing the cytoplasmic membrane potential [17] and inhibits plasmid replication *via* inhibition of DNA gyrase [18]. Furthermore, indole is implicated in many stress responses. There are conflicting reports of indole either improving [19] or reducing [16] *E*. *coli* survival under extreme acid challenges. Under alkaline conditions TnaA expression is induced and the consequent tryptophan degradation gives rise to acidic products, including indole [20, 21]. High temperature (50°C) has been reported to increase indole production by *E*. *coli* and this has been proposed to enhance survival [22].

Indole is also implicated in antibiotic resistance. It can induce multidrug exporters, such as *acrD* and *mdtE*, to increase antibiotic tolerance of *E*. *coli* [23]. Under conditions of antibiotic stress, indole may be secreted by a sub-population of resistant bacteria to aid the growth of their isogenic, non-resistant siblings [24]. In addition, indole affects bacterial persistence; the phenomenon by which genetically sensitive cells survive in the presence of a high concentration of an antibiotic. However, it is still a subject of debate whether indole increases [25, 26] or decreases [27, 28] the frequency of persister cells.

As summarised above, there have been many reports on the causes and consequences of indole production under a variety of conditions. However, they can be difficult to compare because of the diversity of experimental approaches. These include monitoring the effect of adding exogenous indole [16, 18, 23], measuring indole production in response to treatments [24] or comparing the phenotype of a wild-type strain with an indole-negative mutant (*ΔtnaA*) [25]. Some reports, while interesting, are incomplete. For example, Han *et al*. [22] reported that *E*. *coli* produces copious indole at 50°C. They hypothesized that indole thus produced “may lead to a higher survival rate at high temperatures” but provided no evidence to support this. The potential importance of *E*. *coli* survival at high temperature, not least in the food industry [29], prompted us to look more closely at this phenomenon. Here we investigate whether heat does indeed stimulate indole synthesis and, if so, whether this aids survival under heat stress. We find that, although indole can assist cells to withstand heat stress under some growth conditions, the indole synthesis reported by Han *et al*. does not fulfil this role. Our study is illuminated by an analysis of the amount and distribution of TnaA in *E*. *coli* cells during different phases of growth and provides further insight into post-translational regulation of tryptophanase.

## Materials and methods

### Strains and culture conditions

*E*. *coli* BW25113 and BW25113 *ΔtnaA* were obtained from the Keio collection [30]. BW25113 *tnaA-gfp* was from a previous study [31]. Cells were cultured routinely in LB medium (Formedium LB-broth Miller, Hunstanton, UK) at 37°C, with shaking at 120 rpm in a shaking incubator (INFORS HT, Bottmingen, Switzerland) or 200 rpm in a water bath (Grant SS40-D, Cambridge, UK). Independent colonies on stock LB agar plates were picked to inoculate 5 mL fresh LB broth (in 30 mL universal tubes; Thermo Fisher Scientific, Newport, UK) and incubated in the shaking incubator for 16–20 h. Overnight cultures were diluted to OD_600_ = 0.01 to start fresh cultures and cells usually reached exponential growth after 60 min.

### Inhibition of protein synthesis

Chloramphenicol (Sigma, China) stock solution (34 mg·mL^-1^) was added to a stationary phase culture to a final concentration of 34 μg·mL^-1^ and incubated for 1 h before heat treatment. This method has been used in previous studies to stop protein synthesis [32].

### Heat stress

Overnight LB broth cultures (16 h) were centrifuged at room temperature for 10 min at 3,050 x *g* (Eppendorf 5810R, Hamburg, Germany). The pellet was washed with PBS buffer and resuspended in fresh LB medium to OD_600_≈0.1. The resuspension was incubated at 50°C, with shaking at 200 rpm. Exponential phase cultures were grown from OD_600_≈0.01 to OD_600_≈0.1, which usually took 90 min, then transferred to 50°C with shaking at 200 rpm. All heat stressed cultures were 20 mL, in 50 mL conical flasks. OD_600_ was measured using a GeneUant 1300 spectrometer (Uppsala, Sweden). For estimating CFU, cultures were diluted appropriately in PBS buffer and 100 μL of the dilution was spread on LB agar (Formedium LB-agar Miller, Hunstanton, UK) plates. These plates were incubated at 37°C in a static incubator (LTE Scientific IP100-U, Oldham, UK) for 48 h and then for another 24 h at room temperature to detect late appearing colonies [33]. PBS buffer consisted of 8 g NaCl (Fisher Scientific, Loughborough, UK), 0.2 g KCl (Sigma, Germany), 1.78 g Na_2_HPO_4_·2H_2_O (BDH Chemicals, Poole, UK), 0.24 g KH_2_PO_4_ (Fisher Scientific, Loughborough, UK) dissolved in 1 L distilled water.

### Kovacs assay

The Kovacs assay protocol was modified from the method of Darkoh *et al*. [34] in order to detect indole in the range of 10–40 μM as well as the normal sensitivity range of 50–500 μM. 200 μL of culture was centrifuged at 11,337 x *g* (Eppendorf, Minispin Plus, Hamburg, Germany) for 2 min. 100 μL of the supernatant was mixed with 150 μL of Kovacs reagent (5 g *p*-dimetylaminobenzaldehyde, Sigma, Belgium; 75 mL amyl alcohol, Sigma, Germany; 25 mL 37% w/w HCl, Fisher Scientific, Loughborough, UK) and incubated at room temperature for 5 min. The absorbance of the reaction mix was measured at 530 nm using a SpectraMax 190 microplate reader (Molecular Devices, Sunnyvale, USA). The concentration of indole was calculated using a standard curve generated with indole (Sigma, USA) standards dissolved in ethanol.

### Flow cytometry

Samples with OD_600_>0.1 were diluted in ice cold PBS buffer to OD_600_≈0.1. All samples were kept on ice before being analysed in the flow cytometer (Beckman Coulter CyAn ADP, Indianapolis, USA). Fluorescence was excited at 488 nm and measured through an emission filter with bandpass of 530/40. For each sample, 100,000 events were counted at a rate between 1000 and 3000 events per second. Data was processed by R (3.2.5) with flowCore, flowViz and flowDensity packages.

## Results

### *E*. *coli* indole production remains functional at 50°C

If indole production is an integral part of the cellular response to heat stress, both tryptophan import and the indole production enzyme (TnaA) must, of necessity, be active at the relevant temperature. Preliminary experiments suggested that indole production was significantly reduced at 60°C. Thus when a stationary phase culture grown previously at 37°C was supplied with 500 μM extra tryptophan (stock solution 40 mM; Sigma, USA) and shifted to 60°C, only 101±43 μM extra indole was produced after 2 h, much less than produced by the 37°C control culture (352±61 μM) or a culture incubated at 50°C (395±80 μM).

**Fig 1:**
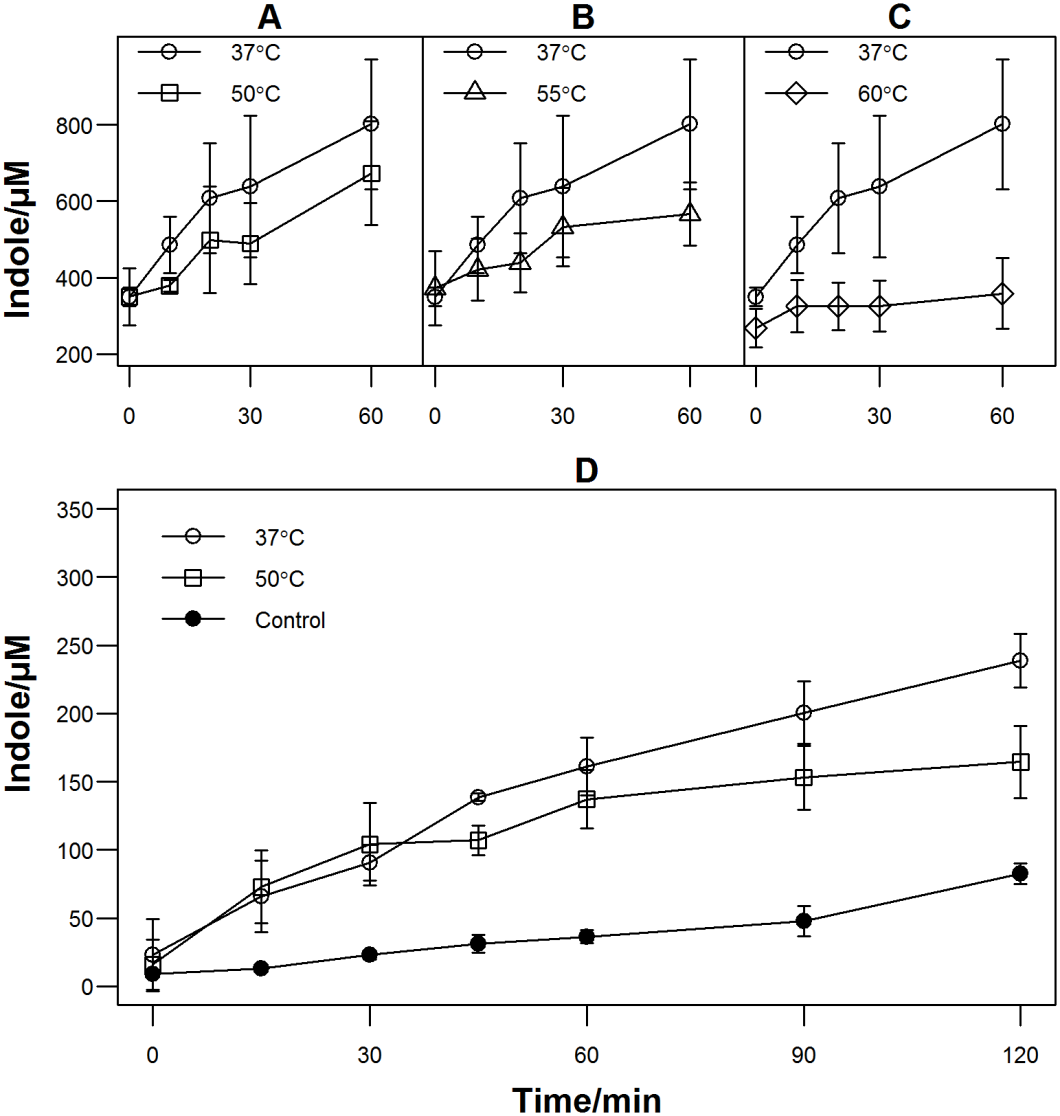
Indole production after incubation at elevated temperatures. Stationary phase cultures of *E*. *coli* BW25113 were treated with chloramphenicol (34 μg·mL^-1^) for approx. 1 h. The cultures were divided into two with one half shifted to (A) 50°C, (B) 55°C or (C) 60°C for 30 min and then returned to 37°C. The other half was kept at 37°C as a control. Tryptophan (500 μM) was added to both halves of the culture and samples were taken at intervals over the next hour for measurement of supernatant indole. (D) Indole production by nutrient-deprived stationary phase cells. Stationary phase cultures were resuspended at OD_600_ = 0.1 in PBS buffer containing 500 μM tryptophan at 37°C or 50°C, or LB medium at 37°C. Samples were taken at intervals for measurement of supernatant indole. Data are the average of at least 3 independent experiments. Error bars show standard deviations.

To identify more accurately the maximum temperature at which indole might plausibly be involved in the heat stress response, we analysed indole production by cells stressed over the temperature range 50–60°C. Our assay for indole production was based on the observation that stationary phase *E*. *coli* in LB broth at 37°C synthesize indole rapidly when the culture medium is supplemented with tryptophan [31]. Stationary phase cultures were treated with chloramphenicol to stop further protein synthesis and were then incubated at an elevated temperature for 30 min. Subsequently, cultures were returned to 37°C and tryptophan (500 μM) was added. Indole synthesis was measured over the next hour using the Kovacs assay. It was clear that 30 min at 60°C (Fig 1C) abolished the ability of cells to make indole, possibly through inactivation of the tryptophanase enzyme (concentrations at all time points significantly less than the control). The concentration of indole in the heat-treated samples did not increase, even after 24 h at 37°C (remaining at the stationary phase basal level of 231±81 μM). Incubation at 55°C impaired but did not eradicate indole synthesis. After 60 min at 37°C the heat-treated samples accumulated less indole than the unstressed control (Fig 1B; differences not significant except at t = 60 min), but reached the same level as the control after 24 h (617±83 μM). After 30 min at 50°C (Fig 1A), indole accumulation was similar to the unstressed control (differences not significant at any time points). We conclude that indole signalling might plausibly be part of the heat stress response up to 55°C, but not at 60°C or above. We decided to use 50°C to stress our cells in the remainder of this work.

### Indole production at 50°C does not aid survival of stationary phase *E*. *coli*

**Fig 2:**
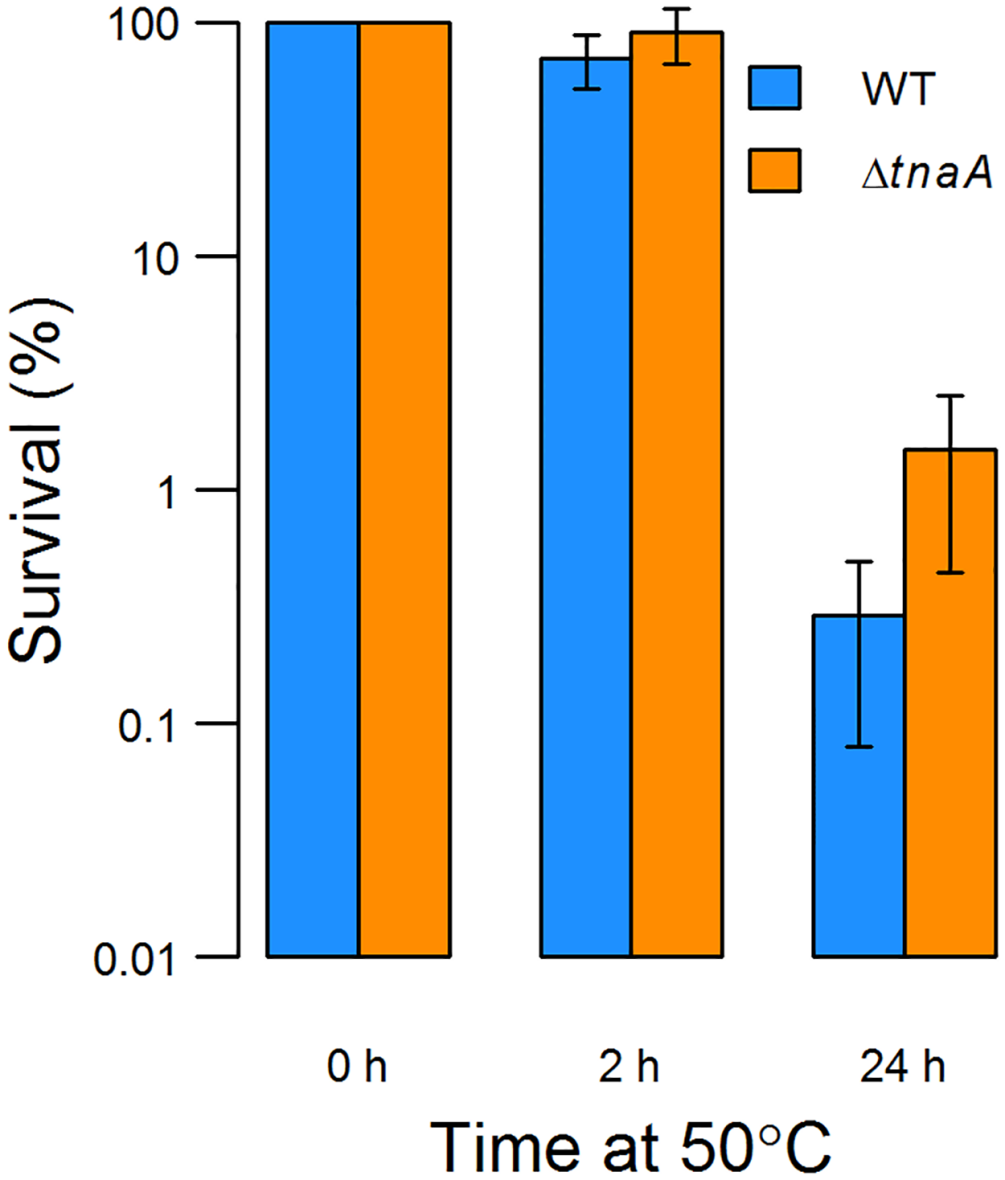
Survival of stationary phase cells at 50°C. An overnight culture was pelleted by centrifugation, resuspended in fresh medium and incubated at 50°C. Survival of wild-type and *ΔtnaA* cells after 2 h and 24 h is shown as a percentage of CFU in each culture at the start of the experiment. The difference is statistically insignificant at t = 2 h but statistically significant at t = 24 h. Data are the average of 6 independent experiments. Error bars show standard deviations.

We repeated the key experiment of Han *et al*. [22] by sub-culturing a stationary phase wild-type *E*. *coli* culture in fresh LB medium (final OD_600_≈0.1) and incubating at 50°C for 24 h (Fig 2). Culture temperature rose to 48°C from room temperature within 6 min. There was no increase in culture density during this time. After 2 h, 70% of cells remained viable, which is similar to the previous report (Han *et al*. observed 93% survival). After 24h, however, more than 99% had been killed, confirming that extended incubation at 50°C is lethal to *E*. *coli*. After 2 h the concentration of indole in the supernatant of the culture was approx. 200 μM and this rose to approx. 300 μM after 24 h. This is consistent with the observation of Han *et al*. [22] that *E*. *coli* produces a substantial amount of indole at 50°C.

Han *et al*. [22] proposed that indole production assists *E*. *coli* survival at 50°C but failed to provide any direct evidence for this. To test this assertion, the survival of a wild-type culture was compared with an indole-negative mutant (*ΔtnaA*) (Fig 2). It was clear that indole production did not improve survival of the wild-type in comparison with the indole-negative strain. If anything, the indole-negative strain showed better survival.

### Growth inhibition is the primary cause of *E*. *coli* indole production at 50°C

In the experiment shown in Fig 2, and in the work of Han *et al*. [22], cells experienced both direct and indirect effects of heat. Direct effects include protein denaturation [35] and alteration in membrane fluidity [36] while the most obvious indirect effect is the abolition of growth. We thought it possible that the abolition of growth, rather than the increased temperature that caused it, might be primarily responsible for indole synthesis. In support of this Fig 1A shows that the addition of tryptophan to chloramphenicol-treated stationary phase cultures is followed by a period of rapid indole synthesis both at 37°C and at 50°C (Fig 1A). To test this proposal further we compared indole synthesis at 37°C and 50°C by cells sub-cultured to OD_600_ = 0.1 in an isotonic buffer (PBS) supplemented with tryptophan (500 μM). Under these conditions cells grow very little (the OD_600_ at 37°C increased by about 30% during the experiment) but they can, potentially, synthesize indole. Indole synthesis occurred in both cultures and, up to 60 min, there was no difference between indole accumulation at 37°C and 50°C (Fig 1D; differences not significant except at times t = 45, t = 90, t = 120 min). At 50°C accumulation slowed after 60 min, possibly due to slow denaturation of tryptophanase. In contrast, when stationary phase cells were sub-cultured to OD_600_ = 0.1 in LB medium at 37°C and allowed to grow, indole production was much lower. Only 30 μM indole accumulated in the first 60 min (Fig 1D), even though the culture density (OD_600_) increased from 0.1 to 0.5 over this period. Between t = 60 and t = 120 min indole synthesis increased as the culture approached the transition to stationary phase when rapid indole production is known to occur [37].

These experiments seem to demonstrate that when *E*. *coli* is not growing, or is growing very slowly, it produces indole from tryptophan regardless of the temperature. What is more, indole production does not require *de novo* expression of TnaA as it occurs even when protein synthesis has been inhibited by chloramphenicol. Therefore, the indole production seen by Han *et al*. [22] at 50°C may not be a direct response to elevated temperature, but rather a response to the associated inhibition of growth. If indole is not produced primarily as a response to elevated temperature, this helps to explain why it gave no benefit for cell survival at 50°C.

### *E*. *coli* tryptophanase (TnaA) content is highly heterogeneous

A possible reason why indole did not improve the survival of stationary phase cells at 50°C (Fig 2) is that these cells are intrinsically stress resistant due to their high σ^S^ level [38] and the fact that they are neither growing nor dividing. These factors may eclipse any potential benefit of indole production. We therefore decided to repeat the heat stress experiment with exponentially growing cells, which have little or no σ^S^ and a greater sensitivity to stress [38]. As a preparatory step in this investigation we used flow cytometry to quantify the TnaA content of exponential phase cells.

**Fig 3:**
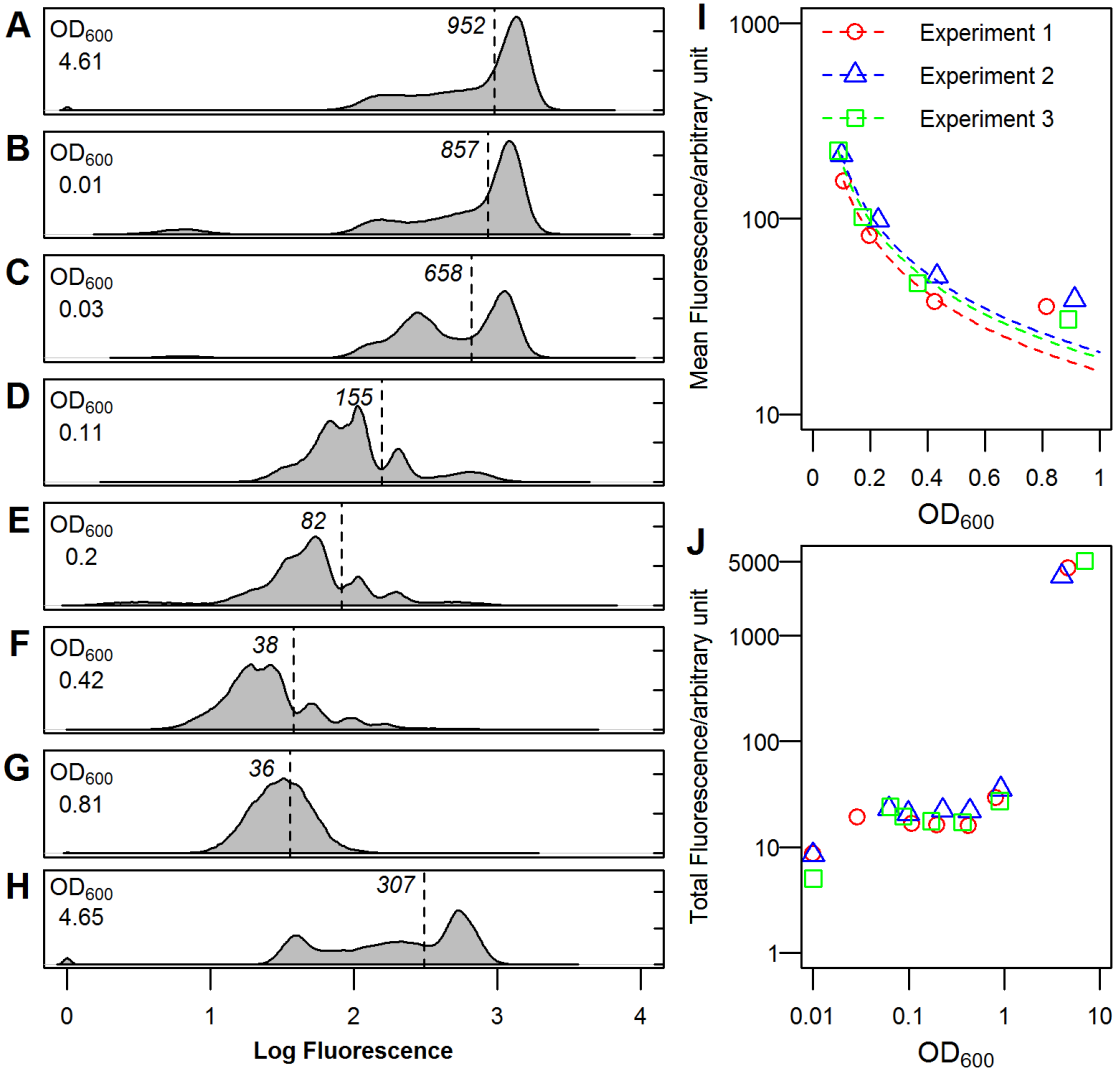
Expression of TnaA-GFP in different phases of growth. (A) overnight culture used for inoculation; (B) immediately after inoculation; (C)-(F) exponential phase; (G) transitional phase; (H) early stationary phase. The mean fluorescence is indicated by a dashed line and shown in arbitrary units. (I) Variation in mean TnaA-GFP fluorescence during exponential phase. Samples from the same replicate are represented in the same colour. The broken lines indicate the expected change in mean cellular fluorescence in the absence of *de novo* tryptophanase synthesis. (J) The total fluorescence of the bulk culture from which samples were taken was estimated by multiplying the mean fluorescence by OD_600_ for each sample, assuming cell density is proportional to OD_600_ in exponential phase [39].

Fig 3 shows the distribution of GFP-labelled tryptophanase (TnaA-GFP) when a 16 h stationary phase culture (panel A) was sub-cultured into fresh LB medium at 37°C (panel B), entered exponential phase (panels C to F) and finally returned to stationary phase (panels G and H). The data are independent of the culture density because the flow cytometer analysed the same number of cells from each sample. In addition to the TnaA-GFP distribution, each panel also displays the mean TnaA-GFP fluorescence for each sample; its position in the distribution is shown by a vertical line. TnaA content displayed striking heterogeneity in stationary phase (panel A) and this heterogeneity persisted until late exponential phase (panel G). Mean cellular fluorescence declined steadily during exponential phase, before increasing sharply during the transition to stationary phase. Fig 3I shows the variation in mean TnaA-GFP fluorescence with culture density in three independent experiments. The broken lines show the expected decline in mean fluorescence in the absence of *de novo* TnaA synthesis during exponential phase. The close correlation between prediction and data up to OD_600_ = 0.4 suggests that the cells were producing very little TnaA during early exponential phase. It is apparent that TnaA synthesis resumed between OD_600_ = 0.4 and 0.8 (Fig 3 panels F-G) as the mean cellular fluorescence remained unchanged despite the increase in culture density. The total fluorescence of each sample (Fig 3J) remained constant throughout exponential phase before increasing sharply at the transition to stationary phase (OD_600_>1). This agrees well with a previous study monitoring TnaA-GFP expression in bulk culture [31]. Thus although cells in exponential phase synthesise almost no TnaA, they do contain enzyme inherited from their ancestors that was synthesised during entry into the previous stationary phase.

### A low concentration of indole aids survival of exponentially growing *E*. *coli* at 50°C

**Fig 4:**
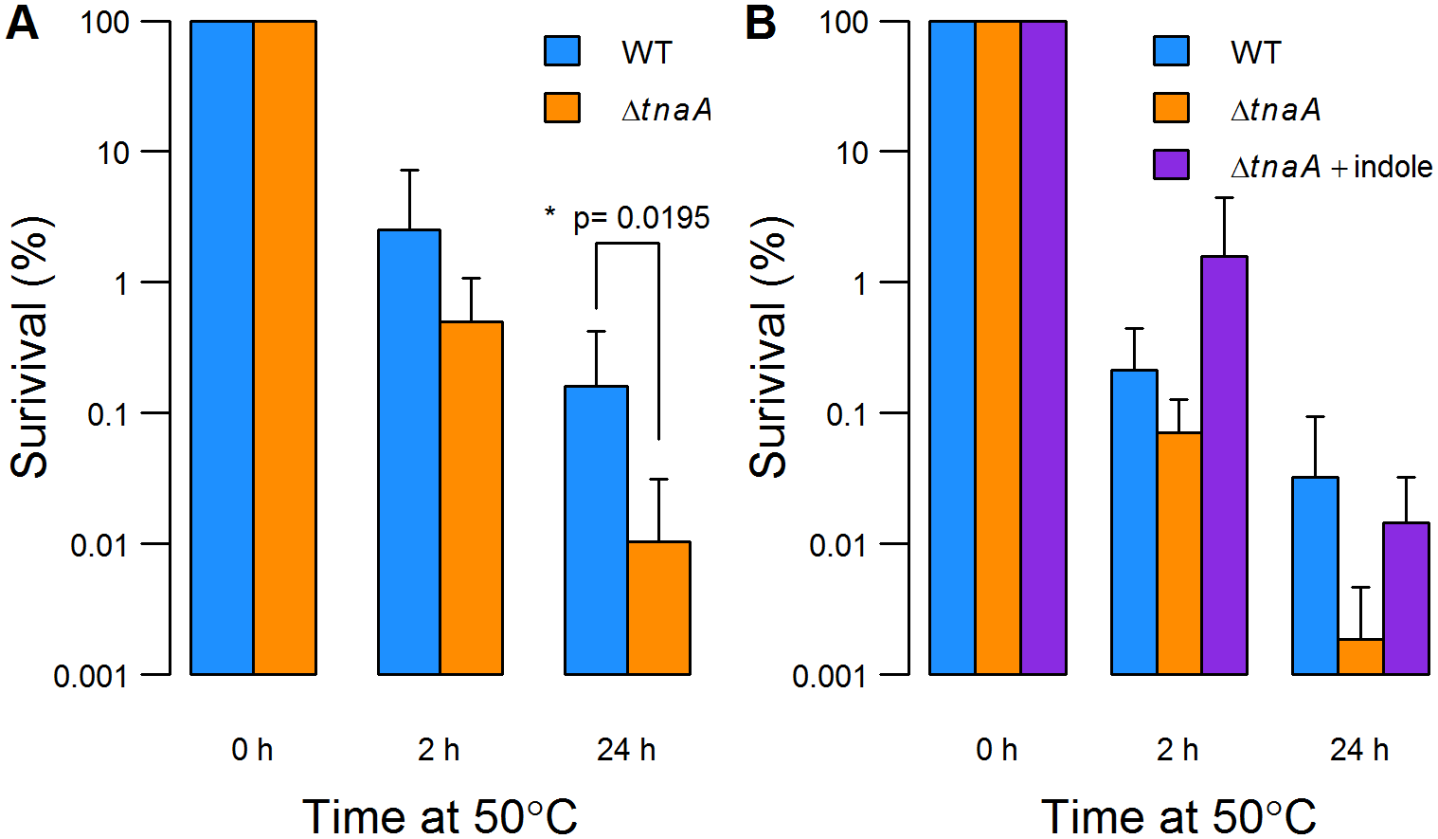
Survival of exponential phase cells at 50°C and the effect of indole supplementation. (A) Exponential phase cultures of wild-type and *ΔtnaA* cells (OD_600_≈0.1) were shifted from 37°C to a 50°C and % survival was measured after 2 h and 24 h. There is a significant difference between the survival of wild-type and *ΔtnaA* at 24 h (t-test: t = 2.3073, df = 12.374 [Welch’s correction], p = 0.0195). (B) To test the effect of indole supplementation, 20 μM indole (dissolved in DMSO) was added to a *ΔtnaA* culture. An appropriate amount of DMSO was added to the control cultures that lacked indole. All data are the average of at least 4 independent cultures. Error bars show standard deviations.

Having established the tryptophanase content of exponentially growing cells, we tested whether an ability to produce indole protects cells from heat stress during this phase of growth. Wild-type and *ΔtnaA* cultures (OD_600_≈0.1) were shifted from 37°C to 50°C and incubated for a further 24h (Fig 4A). At the time of the temperature shift, the cells contained on average about 16% of the TnaA content of stationary phase cells (Fig 3, compare panels A and D). As expected, exponentially growing cells were far more sensitive to heat stress than their stationary phase counterparts. While stationary phase cell survival after 2h at 50°C was approx. 70% (Fig 2), this fell to approx. 1% for exponential phase cells (Fig 4A). Comparing exponentially growing wild-type and indole negative (*ΔtnaA*) cultures, there was no significant survival difference after 2 h at 50°C, but after 24 h the wild-type showed 10-fold greater survival than the *ΔtnaA* mutant. The Kovacs assay was used to measure indole in these cultures. When the cultures were first shifted to 50°C, supernatant indole was undetectable (<10 μM). Over the course of the experiment, the wild-type culture accumulated approx. 20 μM indole, far less than the 300–500 μM that could theoretically have been produced if all of the free tryptophan in LB medium had been converted [37, 40]. When the experiment was repeated using a *tnaA-gfp* fusion strain, no *de novo* TnaA expression was detected at 50°C, either by bulk culture fluorescence measurement or flow cytometry (data not shown). Thus indole detected in supernatant of the wild-type culture at 50°C was made by TnaA already present in the cells at the time of the temperature shift.

We attempted chemical complementation of the *ΔtnaA* culture by adding indole (20 μM) at the start of the heat stress (Fig 4B). Since the solvent used to dissolve indole (DMSO, 10% v/v) might have affected *E*. *coli* survival at 50°C, an appropriate concentration of DMSO was added to the indole-free controls. In the absence of added indole, the difference in survival between the wild-type and *ΔtnaA* strains was approx. 10-fold. The addition of 20 μM indole substantially improved survival of the mutant strain, making it indistinguishable from the wild-type. It therefore appears the low concentration of indole in the culture supernatant is sufficient to account for the greater survival of exponentially-growing wild-type cells at 50°C.

## Discussion

A key objective of this work was to test the assertion by Han *et al*. [22] that indole production in *E*. *coli* is triggered by high temperature and that this leads to improved survival under heat stress. We corroborated the observation of Han *et al*. [22] that *E*. *coli* makes a substantial amount of indole when stationary phase cells are sub-cultured into fresh LB medium at 50°C. However, when the survival of these cells was compared with an indole non-producing strain under the same conditions we found that there was no survival advantage for the indole producer (Fig 2).

Further investigation cast doubt on the assumption that indole synthesis observed by Han *et al*. [22] had been triggered directly by the high temperature. Rather we found that high level indole synthesis occurs whenever stationary phase cells are provided with tryptophan under conditions where they cannot enter exponential phase. Thus high level indole synthesis was seen (i) when tryptophan was added to stationary phase culture at either 37°C [31] or 50°C and (ii) when stationary phase cells were sub-cultured into PBS buffer plus tryptophan at either 37°C or 50°C (Fig 1D). This suggests that the indole synthesis observed by Han *et al*. [22] occurred not because of the high temperature *per se* but because the high temperature prevented growth. If high level indole synthesis was not a specific response to heat, this explains the otherwise paradoxical observation that it did not aid wild-type *E*. *coli* survival under heat stress (Fig 2).

Stationary phase cells are inherently stress resistant so we extended our investigation to exponentially-growing cells. In this case we did find a protective role for indole; after 24 h at 50°C there was a 10-fold survival advantage for wild-type *E*. *coli* over an indole non-producing mutant (Fig 4A). We were surprised that the supernatant of the wild-type culture accumulated only 20 μM indole during this period. However, the addition of 20 μM indole to an indole non-producing (*ΔtnaA*) culture restored the wild-type survival level (Fig 4B), demonstrating the low concentration of indole is both necessary and sufficient for heat stress protection. This concentration is much lower than the “physiologically relevant” concentrations of 100–1,000 μM normally quoted in the literature. However, there is one study showing that *E*. *coli* pre-incubated with 15 μM indole for 20 h had improved survival after ofloxacin treatment [25]. The mechanism by which a low concentration of indole protects the cells is unknown, but may involve the induction of heat shock proteins. Indole has been reported to induce chaperones and proteases, such as DnaK and Lon in the soil bacterium *Pseudomonas putida* and the diesel-degrading bacterium *Acinetobacter oleivorans* DR1 [41, 42].

Our flow cytometry analysis demonstrates a highly heterogeneous distribution of TnaA-GFP among cells in both stationary and exponential phase. Assuming a simple correlation between fluorescence and enzyme content, an early stationary phase culture (Fig 3H) appears to contain high and low fluorescence subpopulations whose tryptophanase content differs by more than 10-fold. Even during exponential phase, when the distribution becomes more uniform, high fluorescence sub-populations are still visible in most samples. The heterogeneous distribution of TnaA in exponentially growing cells makes it unlikely that all contribute equally to indole production. Our observation that the addition of 20 μM indole to the culture medium restores the wild-type level of heat stress resistance to tryptophanase deficient cells shows that the benefits of indole are not restricted to cells that produce it. This hint of altruism resonates with a previous study showing altruistic indole production underpinning the evolution of antibiotic resistance [24].

Finally, this work sheds further light on post-translational regulation of TnaA. Wild-type *E*. *coli* cells grown to stationary phase in LB medium contain a substantial amount of tryptophanase. We know that this enzyme is active because rapid indole synthesis results when tryptophan is added to the stationary phase culture [31], or if the cells are sub-cultured into tryptophan-containing PBS buffer (Fig 1D). However, when the cells are sub-cultured into fresh LB medium, very little indole is produced. Flow cytometry shows that the enzyme is still present as the cells enter exponential phase, suggesting it must have been inactivated. This has close parallels with the observation of Li and Young [13] who showed that a range of carbohydrates, such as glucose and arabinose, inhibit TnaA activity by an indirect and ill-defined mechanism. In both cases, deactivation of tryptophanase appears to be associated with increased metabolic activity.
